# Deep Learning-Based Computer-Aided Diagnosis (CAD): Applications for Medical Image Datasets

**DOI:** 10.3390/s22228999

**Published:** 2022-11-21

**Authors:** Yezi Ali Kadhim, Muhammad Umer Khan, Alok Mishra

**Affiliations:** 1 Department of Modeling and Design of Engineering Systems (MODES), Atilim University, Ankara 06830, Turkey; 2 Department of Electrical and Electronics Engineering, Atilim University, Incek, Ankara 06830, Turkey; 3 Department of Mechatronics Engineering, Atilim University, Incek, Ankara 06830, Turkey; 4 Department of Software Engineering, Atilim University, Incek, Ankara 06830, Turkey; 5 Informatics and Digitalization Group, Molde University College—Specialized University in Logistics, 6410 Molde, Norway

**Keywords:** deep learning, CNN, auto-encoder, ant colony optimization, COVID-19, brain tumor

## Abstract

Computer-aided diagnosis (CAD) has proved to be an effective and accurate method for diagnostic prediction over the years. This article focuses on the development of an automated CAD system with the intent to perform diagnosis as accurately as possible. Deep learning methods have been able to produce impressive results on medical image datasets. This study employs deep learning methods in conjunction with meta-heuristic algorithms and supervised machine-learning algorithms to perform an accurate diagnosis. Pre-trained convolutional neural networks (CNNs) or auto-encoder are used for feature extraction, whereas feature selection is performed using an ant colony optimization (ACO) algorithm. Ant colony optimization helps to search for the best optimal features while reducing the amount of data. Lastly, diagnosis prediction (classification) is achieved using learnable classifiers. The novel framework for the extraction and selection of features is based on deep learning, auto-encoder, and ACO. The performance of the proposed approach is evaluated using two medical image datasets: chest X-ray (CXR) and magnetic resonance imaging (MRI) for the prediction of the existence of COVID-19 and brain tumors. Accuracy is used as the main measure to compare the performance of the proposed approach with existing state-of-the-art methods. The proposed system achieves an average accuracy of 99.61% and 99.18%, outperforming all other methods in diagnosing the presence of COVID-19 and brain tumors, respectively. Based on the achieved results, it can be claimed that physicians or radiologists can confidently utilize the proposed approach for diagnosing COVID-19 patients and patients with specific brain tumors.

## 1. Introduction

Ever since the development of the first known “expert system” in medicine, researchers have been striving to explore the possibilities of engaging artificial intelligence to solve medical problems of various natures [1]. The advent of modern computers paved the way for a completely new domain that emerged as a combined struggle of medical personnel and computer scientists, known as computer-aided diagnosis (CAD) [2]. The first commercial CAD system was approved by the US Food and Drug Administration (FDA) for mammography in 1998 [3]. Since then, several CAD systems have been developed for analyzing the chest, brain, heart, colon, and kidney, among many other organs, based upon conventional projection radiography, ultrasound [4], computed tomography (CT), and magnetic resonance imaging.

The modern concept of the CAD is to help physicians, pathologists, or radiologists in decision-making. However, researchers in the 1960s and 1970s had a different perspective [5,6]. The original intention was to replace humans with computers for detecting abnormalities, as many considered them superior to perform specific tasks. However, in the following years, computers could not prove their supremacy, probably due to insufficient computational capability and the unavailability of advanced image-processing and image-interpretation techniques. It helped to change the notion from computers being the diagnosticians to the assistants of a physician for decision-making.

In recent years, due to the availability of better computational tools and processors, some investigators have made impressive progress toward the development of automated computer-aided diagnosis tools [7]. The purpose is to develop error-prone diagnostic methods that can achieve a higher level of diagnosis results than the average level achievable by physicians. A significant portion of the CAD systems depends upon the computational analysis of the images to deduce the conclusion. Modern computational tools, particularly deep learning methods, have been effectively utilized for the analysis to obtain promising results [8]. This study employs more recent deep learning methods to encounter the most explored problems in medicine, COVID-19, and brain tumors.

COVID-19 broke out in late 2019 and has not only claimed many lives all around the world, but also impacted many economically and socially. Detecting COVID-19 at the earlier stages can prevent the loss of many precious lives. The nucleic acid detection kit (TR-PCR) and nucleic-acid sequencing are among the most commonly used diagnostic procedures for detection [9]. However, the TR-PCR approach requires at least 8 h to produce test results, while the sequencing takes much longer. By delaying the prompt diagnosis of the afflicted patients, the transmission of this contagious disease can increase at an astonishing rate. Radiologists have utilized CT and chest X-ray (CXR) for lung diseases equally. Many researchers have utilized deep learning technology to diagnose COVID-19 based on CT and chest X-ray (CXR) [10,11,12]. Due to their portability, fewer storage requirements, and availability, deep learning methods are more inclined towards chest X-ray (CXR) over CT for COVID-19 diagnosis.

Brain tumors are also considered among the most explored diagnostics and have seen unprecedented success due to deep learning methods [13]. A brain tumor caused by abnormal cell development that impairs the nervous system’s activities is classified as either malignant or benign [14]. Radiologists widely use MRI for tumor diagnosis. Among all brain tumors, meningioma, glioma, and pituitary are considered the most common types; about 33% of all brain tumors are gliomas [15]. Brain tumor is considered one of the most challenging medical research problems as it exhibits high shape, size, and intensity variations.

Though deep learning methods have achieved promising results in computer-aided diagnosis in recent years, their performance still has much room for improvement. The most notable challenges are the development of advanced segmentation approaches, advanced feature extraction and selection approaches, and better classification approaches. In this study, the main emphasis was on selecting the most effective and essential features based on the ant colony optimization algorithm. Selecting the most prominent features helped improve results by at least 10 percent for both datasets. The main contributions of this paper are four-fold:

- The extraction of features from the image dataset using auto-encoder and well-known pre-trained CNNs, e.g., ResNet and AlexNet;
- The selection of the most notable features using ACO to enhance accuracy;
- A generic framework is proposed that can work on multiple datasets, such as MRIs and X-rays;
- The evaluation of the proposed classification model against the current baseline diagnostic models.

The remainder of this paper is organized as follows: the related work is presented in Section 2; the datasets and their pre-processing, methodologies, deep learning techniques, and the proposed feature selector are described in Section 3; and the results are presented and analyzed in Section 4. Then, in Section 5, some discussions are made. Finally, the conclusion and future recommendations are presented in Section 6.

## 2. Related Works

The adaptation of deep learning methods to perform automated CAD for brain tumors and COVID-19 has been astonishing [16,17]. Deep convolutional neural networks are compelling neural network architectures that can produce promising results when applied for image analysis, pattern recognition, and other purposes. For medical image analysis primarily used for diagnostics, the accuracy of the prediction is vital. The prediction based upon image dataset generally depends upon segmentation and pre-processing, feature extraction and selection, and classification [18].

### 2.1. Segmentation and Pre-Processing

The first stage in the CAD process is gathering the relevant data and executing their quality assessment [18]. After the initial screening, all corrupted images are removed from the dataset. After the attainment of the dataset, a large group of data is distributed into several subsets based upon some uniformity conditions through a process known as segmentation. It is regarded as one of the essential tasks in medical image processing to enhance the already existing information [19]. The wide variety of segmentation techniques can be broadly classified into classical (pixel-based, region-based, edge-based, texture-based) and intelligent (artificial neural network-based, fuzzy logic-based, decision tree-based, genetic algorithm-based) [20]. 

The captured chest X-ray (CXR) and MRI images are mostly considered noisy, inconsistent, and incomplete; therefore, pre-processing these images plays a crucial role in acquiring higher accuracy [21]. Different types of linear and non-linear filters are extensively used for denoising. Filtering is also considered a mean for suppressing unwanted information and enhancing the wanted information. According to their architecture, different deep learning methods have specific input image requirements; thus, image resizing, cropping, normalization, and padding are considered the essential steps in pre-processing [22]. The volume, region, and intensity adjustments are also considered effective pre-processing techniques to improve the execution rate.

### 2.2. Feature Extraction and Selection

Accurate diagnosis is also dependent upon the availability of the large-scale and complex dataset. This also entails the requirement of large memory space and computing power. Feature extraction and selection can be potentially used as a means to remove irrelevant or duplicate information; hence, the time and storage-space requirements can be reduced, which may also lead to the better performance of the CAD systems.

To minimize the information loss and reduce the dimensionality, principal component analysis (PCA) is utilized for the feature selection and extraction [23]. The authors in [24] used a combination of discrete wavelet transform [25] and Gabor filter for feature selection from magnetic resonance images. Some recent work used deep learning techniques for the extraction and selection of features from medical images [22,26,27]. Studies were conducted to extract deep features from brain MRI images using pre-trained networks [28]. Rajpurkar et al. [29] used discriminative chest X-ray (CXR) imaging features to detect pneumonia at different stages through CNN.

### 2.3. Classification

The diagnostic predictions are performed through the central processing module, usually called a classifier. A common problem that exists today is the determination of an appropriate classifier for a given dataset. It is observed that improper classifier selection may substantially reduce the accuracy. Multiple classifiers exist, including those based on neural networks, decision trees, and support vector machines. Recent trends have shown that deep learning-based methods have received much appreciation from investigators for the improved classification on various datasets. In [30], the gray-level co-occurrence matrix is proposed to classify brain tumors as Meningioma, Glioma, and Pituitary based upon CNN. Mustafa R. Ismael et al. [24] proposed a system that integrates statistical features and neural network methods to classify brain cancers in MRI images. The deep convolutional neural network-based multi-grade is presented in [31] for brain tumor classification.

It is typical for a Rician distribution to control the noise in MRI scans. The bendlets system is a second-order shearlet transform with bent elements and is considered an effective tool for sparsely representing images with curve outlines, such as brain MRI scans. An adaptive denoising approach for microsection pictures with Rician noise is proposed in [32] using the bendlet characteristic. This method allows for identifying the curve’s texture and contour as low-frequency components. The Rician noise is clearly recognized to belong to a high-frequency channel, thus making it simple to eliminate without impairing the clarity of the contour.

Alqahtani, Ali et al. developed a novel deep CNN-based framework for chest X-ray analysis [33] that is computationally light and efficient. They used several machine learning classifiers to improve the framework’s discriminating capabilities and suggested a new COV-Net to learn COVID-specific patterns from chest X-rays. The network can assist in the multi-class categorization of two different infection types by utilizing max-pooling procedures.

In [34], soft sensor techniques are used to assess the neurocognitive dysfunctions unique to neurodevelopmental disorders, ADD/HD, and specific learning impairments. The bendlet transform was proposed on the basis of the shearlet transform and used for image processing [35]. K Gopalakrishnan et al. investigated the capacity of several pre-trained DCNN models—Alexnet, Resnet50, GoogLeNet, VGG-16, Resnet101, VGG-19, Inceptionv3, and InceptionResNetV2—using transfer learning to classify diseased brain images [36]. 

In [37], the authors attempted to establish an automated method for categorizing fundus images. Through an ablation study, Tong He et al. evaluated a number of these modifications’ effects on the final model’s accuracy empirically [38]. Samir S. Yadav et al. classified pneumonia using a convolutional neural network (CNN)-based technique on a chest X-ray dataset [39].

In [40], the authors used the deep convolutional neural network to detect COVID-19 from the X-ray imaging system. They introduced the new open-access benchmark that included 13,975 chest X-ray (CXR) images across 13,870 patient case datasets. The three-player knowledge transfer and concentration framework counting a pre-trained appearing network that extracts the chest X-ray (CXR) imaging features from a large scale of lung disease CXR images is presented in [41]. The deep learning based on the DarkNet model is used to classify the binary and multi-classes cases of COVID-19 [42]. In this model, YOLO was employed for the real-time object detection. A rapid and valid method [43] diagnoses COVID-19 with an artificial neural network. They used and tested ten various CNNs to distinguish infection of COVID-19 cases from non-COVID-19.

The existing deep learning diagnostic models use CT, CXR, or MRI images for the detection of brain tumors and COVID-19. The performance indicators of these models indicate that there is still massive room for improvement. In this study, the main emphasis is on selecting the most effective and important features based on ant colony optimization (ACO). The obtained features are then processed through state-of-the-art deep learning classifiers to perform an accurate diagnosis. This paper also strives to develop a generic framework that can work on multiple datasets, such as MRI and CXR.

## 3. Material and Methods

In this study, for detecting brain tumors and COVID-19, the features from the image datasets are obtained either through pre-trained CNN or Auto-encoder. These methods are implemented separately and combined with feature selection methods. The proposed framework tends to work with datasets of different natures. To prove its efficacy, it is tested on two different medical image datasets based on MRI and CXR to detect brain tumors and COVID-19. Both datasets have been used widely by the research community in the recent past.

### 3.1. COVID-19 Dataset

**Figure 1:**
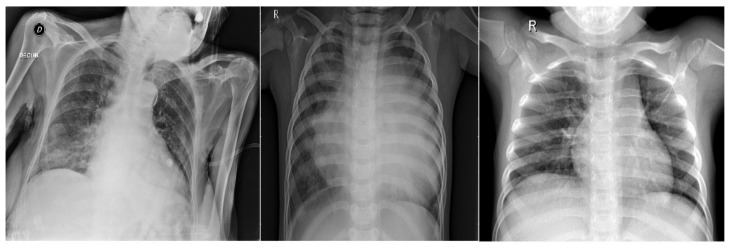
Three classes of COVID-19 dataset: (**left**) COVID-19, (**middle**) Pneumonia, and (**right**) Normal.

The coronavirus strain COVID-19 (coronavirus disease 2019) causes the severe acute respiratory syndrome coronavirus 2 (SARS-CoV-2). The first case was reported in Wuhan, China, in late December 2019, before spreading worldwide [44]. The dataset contains 6432 X-ray images of varying sizes [45]. The distribution for the training-testing is defined as 80-20, consisting of three classes: COVID-19, pneumonia, and normal. The actual number of training images for COVID-19, pneumonia, and normal is defined as 460, 3418, and 1266, respectively. For testing, 116 COVID-19, 855 pneumonia, and 317 normal images are selected. Figure 1 shows a sample from each class for the COVID-19 dataset.

### 3.2. Brain Tumor Dataset

**Figure 2:**
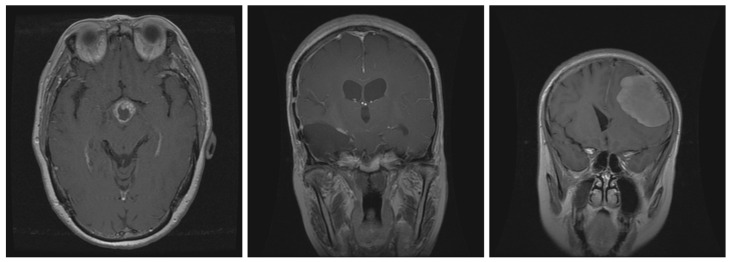
Three classes of brain tumor dataset: (**right**) Meningioma, (**middle**) Glioma, and (**left**) Pituitary Brain Tumors.

The figshare brain tumor MRI dataset is publicly available and is commonly used for classification evaluation. The involved dataset was acquired from Nanfang Hospital, Guangzhou, China, and Tianjin Medical University General Hospital, China, between 2005 and 2010 [46]. This dataset includes 3064 T1-weighted contrast-enhanced images obtained from 233 patients. Three different types of brain tumor are considered in this dataset: meningioma (708 slices), glioma (1426 slices), and pituitary (930 slices). This study defines the distribution between train and test datasets as 80-20. It means that meningioma has 566 slices for training the model and 142 slices for testing, glioma has 1141 slices for training and 285 slices for the testing, and pituitary has 744 slices to train the model and 186 slices for evaluation. Figure 2 represents the brain dataset samples for the three classes.

### 3.3. Deep Learning Techniques

#### 3.3.1. Convolutional Neural Network

This study combines pre-trained CNNs with ant colony optimization (ACO) and some classifiers for image classification. In the first stage, the pre-trained model is applied to extract important features from the image. AlexNet is one of the several pre-trained classifiers involved in this study, and is known as a common model in image classification studies. The study aims to extract high-level and sensitive features from input images. The CNN model realizes human vision technology. Nowadays, CNN is widely used in several computer vision problems as it implements the shared weight technique to reduce the computation rather than the fully connected approach. AlexNet consists of several layers: a convolution layer, pooling layer, and a fully connected layer. The extracted features differ from one classifier to another:

- Alexnet (4096) features.
- Googlenet (1000) features.
- Resnet 50 (2048) features.
- Densenet 201 (1920) features.

#### 3.3.2. Auto-Encoders

An auto-encoder is a neural network that is trained to replicate its input at its output. Auto-encoders can be used as tools to learn deep neural networks. Training an auto-encoder is unsupervised in the sense that no labeled data are needed. The training process is still based on the optimization of a cost function. The cost function measures the error between the input x and its reconstruction at the output $\hat{x}$.

An auto-encoder is composed of an encoder and a decoder. The encoder and decoder can have multiple layers, but for simplicity consider that each of them has only one layer [47,48].

If the input to an auto-encoder is a vector $x\in R^{D_{x}}$, then the encoder maps the vector x to another vector $z\in R^{D^{\left(1\right)}}$, as follows:

(1) $$z=h^{\left(1\right)}\left(W^{\left(1\right)}x+b^{\left(1\right)}\right)$$

where the superscript (1) indicates the first layer. $h^{\left(1\right)}:R^{D^{\left(1\right)}}\to R^{D^{\left(1\right)}}$ is a transfer function for the encoder, $W^{\left(1\right)}\in R^{D^{\left(1\right)}\times D_{x}}$ is a weight matrix, $b^{\left(1\right)}\in R^{D^{\left(1\right)}}$ is a bias vector, and $D_{x}$ is the dimensional of layer *x*; R is real values. Then, the decoder maps the encoded representation *z* back into an estimate of the original input vector, *x*, as follows:

(2) $$\hat{x}=h^{\left(2\right)}\left(W^{\left(2\right)}z+b^{2}\right)$$

where the superscript (2) represents the second layer. $h^{\left(2\right)}:R^{D_{x}}\to R^{D_{x}}$ is the transfer function for the decoder, $W^{\left(1\right)}\in R^{D_{x}\times D^{\left(1\right)}}$ is a weight matrix, and $b^{\left(2\right)}\in R^{D_{x}}$ is a bias vector.

### 3.4. Ant Colony Optimization Algorithm for Feature Selection

One of the interesting behaviors in nature for finding food, which has a high intelligence nature, is the behavior of ants. Ants have a clever way of finding optimal food and reducing their output error in finding the shortest way to it. In this paper, the behavior of ants is first introduced and a modified ant colony optimization for the feature selection method is introduced based on it. In the second phase, the deep learning and auto-encoder-based method are used for classifying the COVID-19 disease and brain tumor data [49].

The ant colony optimization algorithm was proposed in 1990 by Marco Dorigo as an innovative method for solving hybrid optimization problems [49]. This algorithm is derived from the actual behavior of ants to find food along the shortest path [50]. Each ant leaves a chemical material called pheromone along its path of finding food, and other ants choose the shortest path using these previously secreted pheromones. This algorithm is very useful for solving non-deterministic polynomial (NP) problems and is used in problems such as itinerant vendors, scheduling problems, vehicle routing problems, etc. [51].

To solve any NP problem using the ant colony algorithm, the following must be specified:

- First, you have to turn the problem into a graph, including nodes and edges.
- See distance nodes (η) are raised and specified.
- A possible solution is created according to the problem.
- The pheromone update rule is used to determine the effective edges in achieving the best answer.
- The probabilistic transition rule is used to find the next node [52].

Several different implementations of ant colony algorithms such as Ant System, Max-Min Ant System, and Ant Colony System have been proposed; the main difference of these methods is in the pheromone update formula [52]. The method of implementing the proposed algorithm is described. Feature selection will be based on the ant colony method, with two methods of measuring feature-class (FC) and feature-feature (FF).

#### 3.4.1. Working Method of the Proposed Model

**Figure 3:**
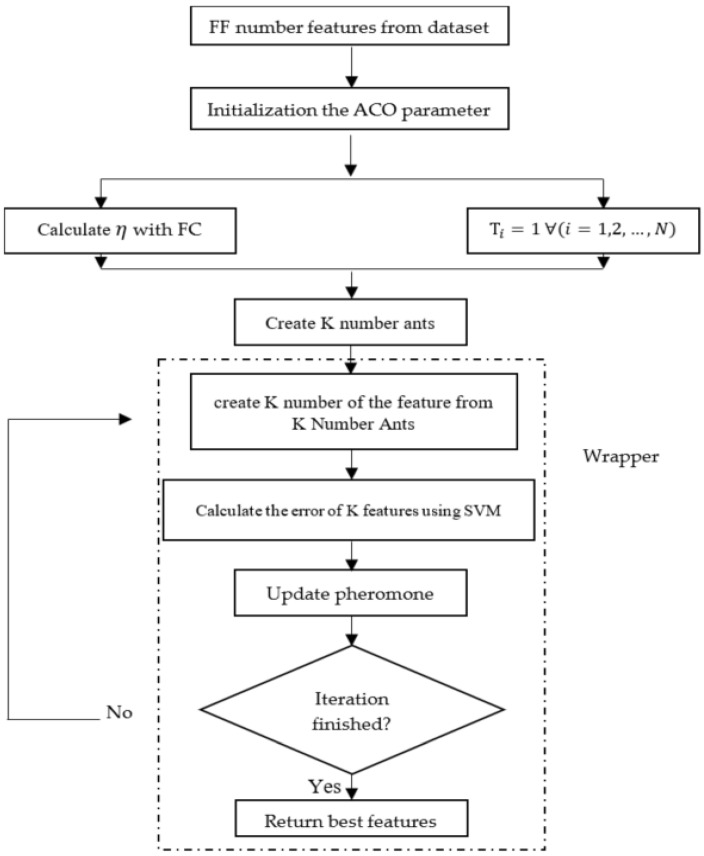
Working method of the proposed feature selection model.

The method of implementing the algorithm is shown in Figure 3. 

In this flowchart, ${Τ}_{i}$ is the pheromone value in ith iteration.

First, a graphical model of all the features in the S dataset is introduced. Attributes are considered nodes and all nodes are interconnected. Then, τ, η, the number of ants, and number of repetitions must be determined [53]. The value of τ is known as the pheromone trail, and at the beginning of the algorithm its values for all attributes are a fixed number of one by default. The value of η is known as heuristic information and is equal to the inverse of the distance between the properties [54]; in this article this distance will be set based on the two methods FC and FF.

After determining the initial values, the algorithm is applicable. In each iteration, the ant is first randomly placed on a node. To determine the next ninety, the law of transfer is used, which is shown in Equation (3):

(3) $$\;P_{i}^{k}\left(t\right)=\frac{{\left|{\;\tau }_{i}\left(t\right)\right|}^{\alpha }\cdot {\left|\eta_{i}\left(t\right)\right|}^{\beta }}{\sum_{u\in j^{k}}{\left|{\;\tau }_{i}\left(t\right)\right|}^{\alpha }.\;{\left|\eta_{i}\left(t\right)\right|}^{\beta }}\;if\left(q>q_{0}\right)$$

$$j=\underset{u\in j^{k}}{\max}\;(\tau_{i}{\left(i\right)}^{\alpha }.\eta_{i}{\left(i\right)}^{\beta })\;if(q<q_{0})$$

The values of *α* and *β* are determined to make the values of *τ* and η more effective. $j^{k}$ is a set of traits that the ant has not yet met, and the trait that the ant has seen before is zero. The parameter $q_{0}$ is very important in determining the choice of both greedy and probabilistic methods, and the value of *q* is a random number between zero and one.

After the *n* ant has completed the node scan, the amount of pheromone obtained from the scan should be updated according to Equation (4):

(4) $$\tau_{i}\left(t+1\right)=\left(1-\rho \right)\tau_{i}\left(t\right)+\sum_{i=1}^{n}∆\tau_{i}^{k}\left(t\right)$$

The value of *ρ* must be determined to reduce the effect. $∆\tau_{i}^{k}$ is the inverse of the error obtained for the Wrapper method and is equal to the average number of nodes selected for the Filter method [53,54].

#### 3.4.2. Criteria for Distance and Similarity of Features

The relationship between two random variables is divided into two categories: linear, and nonlinear. The most famous method for calculating linear variables is the correlation coefficient formula. In [55], the authors used the entropy method and information theory to calculate nonlinear variables. The problem with the correlation coefficient method is that it is inefficient on non-numerical and batch data, while the entropy method works well [55].

Entropy or irregularity criteria are used to measure the uncertainty of a discrete or continuous random variable. The entropy or *H(X)* of the discrete random variable $X=\left(x_{1},x_{2},\ldots ,x_{n}\right)$ is calculated from Equation (5).

(5) $$H\left(X\right)=-\sum_{i=1}^{n}p\left(x_{i}\right)\log\left(p\left(x_{i}\right)\right)$$

where $p\left(x_{i}\right)$ is the probability of $\;x_{i}$ occurring on the whole set.

Calculate the entropy of two discrete random variables *X* and *Y* with dimensions’ n according to Equation (6).

(6) $$H\left(X,Y\right)=-\sum_{i=1}^{n}\sum_{j=1}^{n}p\left(x_{i},y_{i}\right)\log\left(p\left(x_{i},y_{i}\right)\right)$$

Equation (7) is used to calculate the conditional entropy of *X* to condition *Y*.

(7) $$H\left(X|Y\right)=-\sum_{i=1}^{n}\sum_{j=1}^{n}p\left(x_{i},y_{i}\right)\log(p(x_{i}|y_{i}))$$

The purpose of the above formulas is to calculate the information factor (IF). To examine how interdependent the two variables are, the IF criterion is used, which is in accordance with Equation (8):

(8) I(X,Y)=H(X)−H(X|Y)

**Figure 4:**
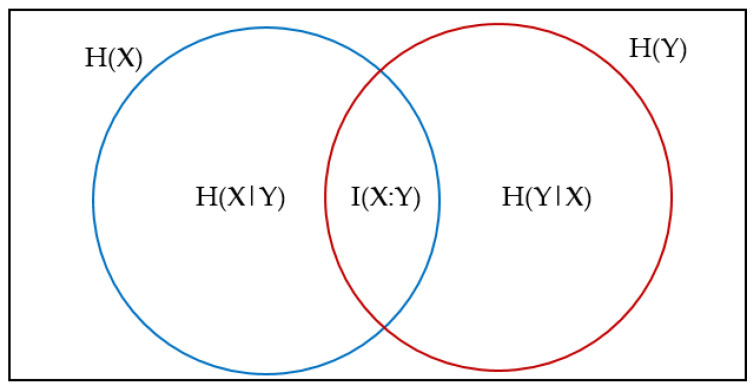
Relationship between information coefficient and entropy.

If the value of IF becomes zero, it means that the two variables are independent, and the higher this value, the more *X* and *Y* are dependent [56]. Figure 4 shows the relationship between information coefficient and entropy.

This study uses the normalized form of IG, known as symmetrical uncertainty (SU), which is consistent with Equation (9). The advantage of this formula is the normality of the dependence of the two variables between the range 0 and 1. If the value of SU is close to one, it means that the two variables are dependent, and if it is close to zero, it means that it is independent.

(9) $$SU\left(X,Y\right)=\frac{2\;*\;I\left(X,Y\right)}{H\left(X\right)+H\left(Y\right)}$$

In this paper, two criteria $SU_{FC}$ and $SU_{FF\;}$ are used to calculate η [55].

The $SU_{FC}$ criterion is defined as the dependence of each attribute on the class. The closer this value is to one, the more important that feature will be and it should be selected.

(10) $$\eta_{i}=\frac{1}{1-SU_{FC}}$$

The criterion $SU_{FF}$ means the dependence of two properties on each other. If its value is close to one, it means that the two properties are very similar, so we will be looking to remove one of the features.

(11) $$\eta_{i}=\frac{1}{SU_{FF}}$$

In selecting attributes, we seek to preserve class-related attributes and remove duplicate or trivial attributes. The goal is to select features with $SU_{FC}$ higher and $SU_{FF\;}$ lower [57].

### 3.5. Dataset Pre-Processing

**Figure 5:**
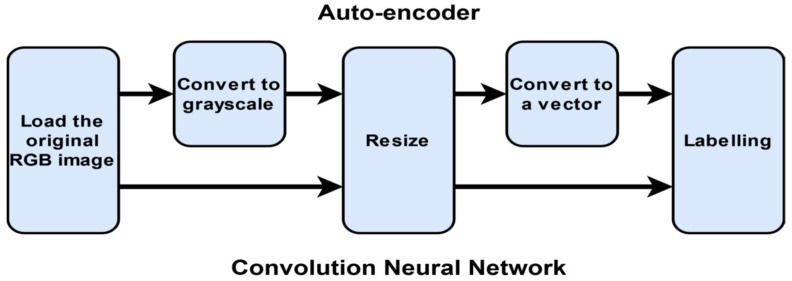
Image dataset pre-processing steps.

Pre-processing image datasets is an essential step that improves the quality of the features, hence diagnosis prediction. The pre-processing steps for CXR and MRI image datasets are detailed in Figure 5. Either auto-encoder or CNN later uses the processed data for the feature extraction. The RGB images are converted to grayscale when processed through an auto-encoder to reduce the amount of processed data by one-third. Furthermore, for auto-encoder images are resized to 64 × 64 and later converted to a single array (vector) without losing any features and to enhance the training model’s quality. The brain tumor images are available as png files with the size of 512 × 512, whereas the chest X-ray (CXR) images have a jpg format of varying sizes. The mismatch among the input images is fixed by resizing them to a fixed 227 × 227 (Alexnet) or 224 × 224 (GoogleNet, ResNet-50, and DenseNet-201) resolution. 

### 3.6. Overall Architecture of the Proposed Framework

**Figure 6:**
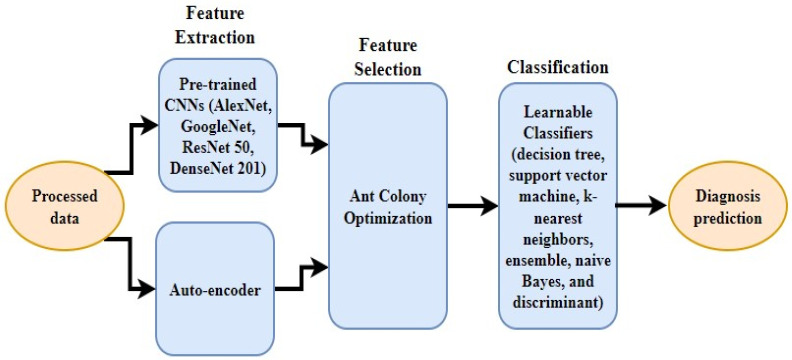
Overall framework of the proposed classification system.

In the proposed framework (Figure 6), features can be extracted either using pre-trained CNNs (AlexNet, GoogleNet, ResNet-50, or DenseNet-201) or auto-encoder. Once features are extracted, the meta-heuristic, ant colony optimization (ACO), is utilized to choose the most effective and prominent features [58].

The main goal of this study is to generate smaller subsets of essential features that help to expedite the search process and select the most optimal features. Afterward, learnable classifiers (decision tree, support vector machine, k-nearest neighbor, ensemble, naive Bayes, or discriminant analysis) can be employed for the diagnosis prediction. These classifiers learn from the features taken from the ACO algorithm and classify them based on the labels of input type. Table 1 shows the characteristics of the chosen classifiers. The initial selection of the classifier can be made based on the trade-off among prediction speed, memory usage, and interpretability.

## 4. Experiments and Results Analysis

In this study, MATLAB2021a is used to execute the proposed method on Intel(R) Core (TM) i7-6500U CPU @ 2.50GHz 2.60 GHz. Many research studies are conducted to investigate the performance of the existing prediction methods. Once done with the pre-processing, the feature selection-extraction is performed, followed by the classification. In the former stage, an auto-encoder/CNN is combined with an ACO algorithm to extract and select the most prominent features from the input training dataset. The second stage employs a range of learnable classifiers to diagnose tumors or COVID-19 accurately.

### 4.1. Evaluation Metrics

The performance evaluation of the proposed framework is based upon the following performance matrices: sensitivity, specificity, precision, accuracy, F1-score, misclassification rate, and Mathews correlation coefficient (MCC). For the confusion matrix, TP represents the true positive, TN represents the true negative, FP represents the false positive, and FN represents the false negative. All the parameters used for the evaluation are listed in Table 2. Among these, accuracy is usually considered the most reliable measure to compare the performance of different classification methods.

### 4.2. Classification Using Learnable Classifiers

The performance of the CAD prediction system depends upon the combination of features and the classifier model. In this study, feature extraction-selection is either done using a combination of auto-encoder + ACO or pre-trained CNNs + ACO. For classification, a broad range of learnable classifiers is selected: decision tree, support vector machine, kernel nearest neighbor, ensemble, naive bayes, and discriminant analysis.

#### 4.2.1. Feature Extraction and Feature Selection Using Auto-Encoder and ACO

In the initial stage, image features from the chest X-ray (CXR) images dataset are extracted using an auto-encoder; whereas most optimal features are selected based on ACO. For training and testing, six different classifiers are chosen to evaluate the performance of the proposed framework. The chest X-ray (CXR) image dataset is used to determine the health status of the patients, such as those affected by COVID-19 or pneumonia or considered normal. Table 3 shows the classification results using the evaluation matrices. The accuracy determined using true values of the test images is considered the most important parameter to evaluate the performance. Support vector machines are able to achieve the highest accuracy of 98.68% among all classifiers, followed by ensemble and discriminant as the second-best classifiers to achieve almost 98% accuracy. The misclassification rate also supports the accuracy results, as the highest MR of 1.31% is obtained using SVM. The NB classifier exhibits the worst performance with the lowest accuracy of 89.28% and an MR of 10.71%.

The MRI image dataset is used to classify three types of brain tumors: meningioma, glioma, and pituitary. Once the image features are selected based on the auto-encoder, ACO searches for the best prominent features. Six different classifiers are used to evaluate the performance of the proposed framework on the MRI image dataset for diagnosing tumors (see Table 3). The classification results demonstrate that the highest classification accuracy of 99.18% is obtained through KNN; likewise, the MR is the lowest, i.e., 0.81%. The worst prediction results are obtained through NB, which achieves the lowest accuracy of 78.62% and the MR of 21.37%. 

#### 4.2.2. Feature Extraction-Selection Using Pre-Trained CNNs and ACO

For the feature extraction of the chest X-ray (CXR) images dataset, several pre-trained CNNs (AlexNet, GoogleNet, ResNet-50, and DenseNet-201) are considered. Once again, the ACO method is employed to select the most prominent image features. The training and testing of the CXR image dataset for the diagnosis of COVID-19, pneumonia, and normal is performed using six classifiers (see Table 1). Table 4 represents the evaluation matrices obtained through all classifiers against four pre-trained CNNs + ACO. The best overall results are obtained when feature extraction is made through ResNet-50 and classification is done through SVM. The highest accuracy of 99.61% is obtained with SVM and an MR of 0.38. The lowest possible accuracy of 94.95% is obtained for AlexNet evaluated against the NB classifier. It is worth mentioning that all the pre-trained CNNs have been able to achieve decent results. 

The MRI image dataset is processed through pre-trained CNNs and ACO to determine the existence of a specific type of brain tumor. The evaluation matrices for classifying brain tumors using six different classifiers are shown in Table 5. It is observable that features extracted using AlexNet, when evaluated against discriminant analysis classifier, produce the best results. The highest classification accuracy of 98.69% is obtained through discriminant analysis, whereas the lowest accuracy of 87.27% is obtained when features using GoogleNet are evaluated against the decision tree classifier.

### 4.3. Performance Comparison with Existing Methods

The comparison analysis is performed for our proposed method with the most prominent methods related to brain tumors and COVID-19. Table 6 shows the classification accuracy results obtained through the selected state-of-the-art methods and our proposed approach using MRI image-dataset. Classification accuracy is a measure to gauge the amount of certainty to perform the correct diagnosis of brain tumor type. For training-testing, a proportion of 80-20 is defined for all the involved methods. The comparison table shows that the proposed method attains superior results in diagnosing brain tumor type when feature extraction, selection, and classification are performed using auto-encoder, ACO, and KNN, respectively.

The same approach is adapted for the COVID-19 image dataset where the proposed method’s performance is compared against the existing methods in terms of accuracy (Table 7). For training-testing, the classification methods, a proportion of 80-20 is defined for all the involved methods. When combined with SVM classifier, the feature extraction-selection using pre-trained CNN (Resnet-50) and ACO produces the highest accuracy index for diagnosing COVID-19, pneumonia, or normal.

(Table 8) compares the work of (ACO) vs. the work of (GA) as a feature selection, and shows an obvious difference in time and accuracy; GA takes twice as long and is less accurate than ACO.

Ant colony optimization reaches the global minimum point faster than the genetic algorithm (GA) as it avoids being trapped in the local minimum. By simulating the intelligent behavior of ants, ACO tries to find optimal solutions to various optimization problems. It has gained considerable interest worldwide because of its advantages, such as simple implementation, small number of parameters, flexibility, etc.

Ant colony optimization has simplicity, flexibility, robustness, scalability, and self-organization. It has few control parameters as compared to a genetic algorithm (GA). The execution of various tasks can be undertaken by individuals simultaneously. Memory space is less utilized by swarm intelligence (SI) compared to GA.

The benefits of ACOs are numerous and there are many stakeholders who obtain advantages from this model of care. The patient community gains a wide number of advantages including improved outcomes, a better quality of care, greater engagement with providers, and an overall reduction in out-of-pocket costs. They have an advantage over the simulated annealing and genetic algorithm approaches of similar problems when the graph may change dynamically; the ant colony algorithm can be run continuously and adapt to changes in real-time [72,73,74].

## 5. Discussion

The advancement in the domain of artificial intelligence has also influenced diagnostics in medical science. This progress has enabled computer scientists to build up CAD tools with the assistance of medical personnel. Physicians frequently use such tools worldwide to perform an accurate diagnosis prediction. Although deep learning methods achieved higher accuracy, their success is highly dependent upon the features and their pre-processing. Today, there exist a plethora of diverse methods; the main problem is the selection of an appropriate method. In this study, we attempted to combine the most suitable methods for feature extraction-selection and classification to attain the highest accuracy using image-dataset.

To prove the effectiveness of the proposed method, two different medical image datasets are employed: CXR for the determination of COVID-19 and MRI for diagnosing brain tumors. Many researchers have evaluated the performance of different existing prediction models for COVID-19 and brain tumors. It is observed that they can achieve accuracy to a certain extent, beyond which further improvement is not made. From [9,69], it is witnessed that the auto-encoder extracted many unimportant image features; thus, it is required that the features should be further enhanced and the best ones should be selected. This study used a unique combination of deep learning techniques and a meta-heuristic algorithm to perform feature extraction and selection. The results reported in Table 6 and Table 7 show that the proposed method outperformed other state-of-the-art methods. This shows that our method is extensive and can be used for different types of medical image datasets. 

In [31], researchers applied the Deep Convolution Neural Network VGG-19 based on the SoftMax classifier, resulting in 94.58% accuracy. This showed that combining the GLCM and Deep Convolution Neural Network VGG-19 leads to highly accurate results, which the authors in [62] obtained for the related comparison of the brain tumor dataset mentioned in Table 6.

For other Covid-19 dataset comparisons in Table 7, when the feature extraction method is not combined with feature selection methods such as Dense Net, we cannot expect results with high accuracy. However, in [41], our study, when we made a combination of the pre-trained Densenet201 with the ACO algorithm, we achieved 98.99% accuracy. 

The method used by GLCM [30,62] has an accuracy is 82.00% and 96.50%. For this type of dataset, a gray level co-occurrence matrix cannot give accurate results from the GLCM features, so GLCM is useful for texture images such as fingerprint and Palmprint images [75]. Also, the method used in [30] used the GLCM, and the result cannot be high, because texture features cannot be used in medical images. However, in [62] they used both GLCM and Pre-Trained VGG-16 CNN, and the combination of the GLCM and Pre-Trained VGG-16 CNN improved the results. That means the use of GLCM only cannot improve the performance of the system. 

In [23], texture feature extraction is used; also, to find the best feature, PCA is used. Principal component analysis feature selection cannot find the best feature from the medical data [76]. Also, there are no combination methods for feature extraction with feature selection employed in the other methods mentioned in Table 6 and Table 7. The ineffective features cannot improve the results. Furthermore, the reason for using these related works is using similar methods either for feature extraction or selection with the same dataset that we used.

Sachdeva et al. [23] recommended PCA based on the intensity and texture features. In this method, PCA finds the eigenvalues of the features and this will tend to find the high eigenvalues in the image. However, in the brain tumor images, some regions of the image are not effective on the high eigenvalues, so the result will contain some mistakes and ultimately the result will be not so accurate.

Cheng et al. [59] used the Bag of Words (BoW) to extract the features from the images and SVM are used in the classification of the images. The BoW is used to recognize the text sources [77,78]. This method for finding the features of medical images is not suitable, and also in [59,60,66] researchers used deep learning with the SVM classifier without the aid of any feature selection function in order to select the effective features and achieve quality results. In [22], a modified deep CNN network was assigned for the dataset to extract the most effective features and classify them with the SVM classifier.

The 2D Discrete Wavelet transform (DWT) and 2D Gabor filter were used in [24]. These feature extraction methods are also useful in the face recognition systems used in [79,80,81].

Capsule networks (CapsNets) were used in [61,71] to extract the features from brain tumor images and Covid-19 images. This method cannot give high accuracy to 2D signals such as medical images and was recently used in speech recognition signals [82]. For 1D signals the performance is better than for 2D signals.

Ghassemi et al. [63] combined the Generative adversarial network (GAN) and ConvNet (random split) to find and classify brain tumor images. Recently, GAN methods were used in a face recognition system [83,84]. Face images have high resolution, and there are more objects on the human face than on tumor images. However, combining with ConvNet can produce high accuracy.

The use of Deep Convolutional Auto-encoder alone cannot produce good results [9,69,70]. Auto-encoder produces more unimportant features from images. These features should be enhanced, and the best ones selected.

Umut Özkaya et al. [64] used CNN with data fusion and they obtained 98.27% accuracy. Compared with other methods, this method can be considered as high performance because data fusion creates robust features.

In Covid-19 images, when the feature extraction method is not combined with feature selection methods such as ANN [65], DNN [67], Random Forest [68], Tailored CNN [40], DenseNet [41], DarkNet-19-based CNN [42], and deep learning [43] we cannot expect highly accurate results.

We implemented a powerful method to extract features from medical image datasets in the proposed study; the deep learning algorithms (CNN, Auto-encoder) produced high-quality results when they extracted features. In addition, the meta-heuristic algorithm was utilized to select the effective features; this combination had a favorable impact. Ant colony optimization was used to select important features, to test its performance.

## 6. Conclusions

Computer-aided diagnostic systems have emerged as an effective tool for performing diagnosis prediction based on medical images. The performance of these systems is challenged by the processing of ‘big data’, which is also vital for accurate diagnosis. This study proposes a novel framework for extracting and selecting features based on deep learning, auto-encoder, and ACO with an intention to select the most prominent image features to reduce the amount of data to be processed. The meta-heuristic algorithm, ACO, is utilized to search for the essential features from the available feature-set to minimize the error rate. The obtained features are then processed through the learnable classifiers to determine the accuracy of the CAD system. The performance of the proposed system is evaluated for two medical image datasets: CXR and MRI. The CXR dataset is used to diagnose the patient’s condition as COVID-19, pneumonia, and normal, whereas, based upon MRI images, the objective is to determine the type of brain tumor (meningioma, glioma, and pituitary). The removal of the minor, redundant, and noisy features produce a significant effect on the accuracy of the overall system. The proposed approach achieves the highest accuracy compared to the other state-of-the-art methods, such as ANN, CNN, CNN with data fusion, Stacked-auto-encoder, Capsule Networks, and DarkNet-19-based CNN, for diagnosing various medical disease datasets. The basic notion of developing the proposed CAD system is assisting the physician. This study’s primary limitation is using the labeled data for supervised learning; hence, this process is not claimed to be completely automated. To exploit the potential of deep learning, the diagnostic prediction must be made using unlabeled data without any human intervention. Further studies will explore the possibility of performing disease prediction without requiring supervision to train the model. 

## Figures and Tables

**Table 1:** The characteristics of classifier types.

Classifier	Prediction Speed	Memory Usage	Interpretability
Decision Tree (DT)	Fast	Small	Easy
Support Vector Machines (SVM)	Medium for linear Slow for others	Medium for linear All others: medium for multiclass, large for binary	Easy for Linear SVM Hard for all other kernel types
Kernel Nearest Neighbor (KNN)	Slow for cubic Medium for others	Medium	Hard
Ensemble	Fast to medium depending on choice of algorithm	Low to high depending on choice of algorithm	Hard
Naïve Bayes	Medium for simple distributions Slow for kernel distributions or high-dimensional data	Small for simple distributions Medium for kernel distributions or high-dimensional data	Easy
Discriminant Analysis	Fast	Small for linear, large for quadratic	Easy

**Table 2:** Evaluation metrics.

Parameter	Abbreviation	Mathematic Expression
Sensitivity, Recall, hit rate, True Positive Rate	(TPR)	$\frac{TP}{TP+FN}=1-FNR$
Specificity, Selectivity, True Negative Rate	(TNR)	$\frac{TN}{TN+FP}=1-FPR$
Precision, Positive Predictive Value	(PPV)	$\frac{TP}{TP+FP}=1-FDR$
Negative Predictive Value	(NPV)	$\frac{TN}{TN+FN\;}=1-FOR$
Accuracy	(ACC)	$\frac{TP+TN}{\left(TP+TN+FP+FN\right)}$
F1 Score	(F1 Score)	$\frac{2\;*\;\left(PPV\;*\;TPR\right)}{\;\left(PPV\;*\;TPR\right)}=\frac{2TP}{\;2TP+FP+FN\;}$
Misclassification rate	(MR)	$\frac{FP+FN}{TP+TN+FP+FN\;}$
Matthews correlation coefficient (MCC)	(MCC)	$\frac{TP\;*\;TN-FP\;*\;FN}{\sqrt{\left(TP+FP\right)\left(TP+FN\right)\left(TN+FP\right)\left(TN+FN\right)}}$

**Table 3:** Auto-encoder with ACO as feature selection-extraction method.

Method	Dataset	ACC	TPR	TNR	PPV	NPV	F1-Score	MR	MCC
DECISION TREE	COVID-19	97.74	88.49	98.63	86.20	98.89	87.33	2.25	0.86
	Brain Tumor	92.82	88.17	94.84	88.17	94.84	88.17	7.17	0.83
SVM	**COVID-19**	**98.68**	**98.05**	**98.73**	**87.06**	**99.82**	**92.23**	**1.31**	**0.92**
	Brain Tumor	96.57	93.19	98.10	95.69	96.95	94.42	3.42	0.92
KNN	COVID-19	97.74	89.18	98.55	85.34	98.97	87.22	2.25	0.86
	**Brain Tumor**	**99.18**	**97.88**	**99.76**	**99.46**	**99.06**	**98.66**	**0.81**	**0.98**
ENSEMBLE	COVID-19	98.21	95.14	98.48	84.48	99.57	89.49	1.78	0.89
	Brain Tumor	95.75	93.95	96.51	91.93	97.42	92.93	4.24	0.90
NAIVE BAYES	COVID-19	89.28	45.45	99.42	94.82	88.73	61.45	10.71	0.61
	Brain Tumor	78.62	59.32	96.54	94.08	71.89	72.76	21.37	0.61
DISCRIMINANT	COVID-19	98.44	98.00	98.48	84.48	99.82	90.74	1.55	0.90
	Brain Tumor	97.06	94.68	98.11	95.69	97.65	95.18	2.93	0.93

**Table 4:** Several pre-trained CNN with ACO as a feature selection method for COVID-19 dataset.

Pretrained CNN with ACO (COVID-19 Dataset)								
Classifiers	ACC	TPR	TNR	PPV	NPV	F1-SCORE	MR	MCC
**Pre-trained CNN (AlexNet) + ACO**								
DECISION TREE	96.11	77.96	97.94	79.31	97.78	78.63	3.88	0.77
SVM	98.99	98.13	99.06	90.51	99.82	94.17	1.00	0.94
KNN	98.68	98.86	98.57	85.34	99.98	92.09	1.31	0.92
ENSEMBLE	98.52	98.98	98.48	84.48	99.91	91.16	1.47	0.91
NAÏVE BAYES	94.95	65.08	99.46	94.82	94.96	77.19	5.04	0.76
DISCRIMINANT	99.53	99.62	99.49	94.82	99.82	97.34	0.46	0.97
**Pre-trained CNN (GoogleNet) + ACO**								
DECISION TREE	97.04	84.82	98.21	81.89	98.54	83.33	2.95	0.82
SVM	98.91	98.11	98.98	89.65	99.82	93.69	1.08	0.93
KNN	98.68	99.00	98.65	86.20	99.91	92.16	1.31	0.92
ENSEMBLE	97.67	95.74	97.82	77.58	99.65	85.71	2.32	0.85
NAÏVE BAYES	98.44	91.37	99.14	91.37	99.14	91.37	1.55	0.91
DISCRIMINANT	98.83	98.09	98.90	88.79	99.82	93.21	1.16	0.93
**Pre-trained CNN (ResNet 50) + ACO**								
DECISION TREE	97.51	91.17	98.06	80.17	99.23	85.32	2.48	0.84
SVM	**99.61**	**99.89**	**99.57**	**95.68**	**99.99**	**97.79**	**0.38**	**0.98**
KNN	98.91	99.03	98.90	88.79	99.91	93.63	1.08	0.93
ENSEMBLE	99.14	99.23	99.07	90.51	99.97	95.02	0.85	0.95
NAÏVE BAYES	99.14	92.00	99.91	99.13	99.14	95.43	0.85	0.95
DISCRIMINANT	99.21	99.32	98.89	99.01	99.21	96.20	0.79	0.95
**Pre-trained CNN (DenseNet 201) + ACO**								
DECISION TREE	97.59	92.92	97.98	79.31	99.40	85.58	2.40	0.85
SVM	98.99	99.04	98.98	89.65	99.91	94.11	1.00	0.94
KNN	98.75	99.37	98.65	86.20	99.90	92.59	1.24	0.92
ENSEMBLE	98.83	99.56	98.73	87.06	99.86	93.08	1.16	0.93
NAÏVE BAYES	99.14	94.11	99.65	96.55	99.40	95.31	0.85	0.95
DISCRIMINANT	98.99	99.57	98.88	92.34	99.80	93.12	1.01	0.94

**Table 5:** Several pre-trained CNN with ACO as a feature selection method for brain tumor dataset.

Pretrained CNN with ACO (Brain Tumor Dataset)								
Classifiers	ACC	TPR	TNR	PPV	NPV	F1-SCORE	MR	MCC
**Pre-trained CNN (AlexNet) + ACO**								
DECISION TREE	91.68	86.09	94.13	86.55	93.91	86.32	8.31	0.80
SVM	98.53	96.33	99.52	98.92	98.36	97.61	1.46	0.97
KNN	98.20	96.29	99.05	97.84	98.36	97.06	1.79	0.96
ENSEMBLE	96.73	93.68	98.10	95.69	97.18	94.68	3.26	0.92
NAÏVE BAYES	89.55	74.79	99.45	98.92	85.48	85.18	10.44	0.79
DISCRIMINANT	**98.69**	**95.87**	**99.92**	**99.95**	**98.12**	**97.89**	**1.30**	**0.97**
**Pre-trained CNN (GoogleNet) + ACO**								
DECISION TREE	87.27	81.76	89.39	74.73	92.74	78.08	12.72	0.69
SVM	95.26	94.85	95.43	89.24	97.89	91.96	4.73	0.89
KNN	94.94	90.15	97.14	93.54	95.55	91.82	5.05	0.88
ENSEMBLE	93.80	91.57	94.71	87.63	96.48	89.56	6.19	0.85
NAÏVE BAYES	91.19	82.67	95.37	89.78	91.80	86.08	8.80	0.80
DISCRIMINANT	96.73	93.22	98.33	96.23	96.95	94.70	3.26	0.92
**Pre-trained CNN (ResNet 50) + ACO**								
DECISION TREE	88.09	79.58	91.94	81.72	90.86	80.63	11.90	0.72
SVM	97.55	98.30	97.24	93.54	99.29	95.86	2.44	0.94
KNN	97.87	95.76	98.82	97.31	98.12	96.53	2.12	0.95
ENSEMBLE	96.24	95.53	96.54	91.93	98.12	93.69	3.75	0.91
NAÏVE BAYES	91.02	79.11	97.93	95.69	88.99	86.61	8.97	0.81
DISCRIMINANT	96.24	94.53	96.97	93.01	97.65	93.76	3.75	0.91
**Pre-trained CNN (DenseNet 201) + ACO**								
DECISION TREE	91.51	86.41	93.70	85.48	94.14	85.94	8.48	0.80
SVM	98.20	96.29	99.05	97.84	98.36	97.06	1.79	0.96
KNN	97.71	95.26	98.81	97.31	97.89	96.27	2.28	0.95
ENSEMBLE	97.22	94.24	98.57	96.77	97.42	95.49	2.77	0.94
NAÏVE BAYES	93.96	87.81	96.87	93.01	94.37	90.33	6.03	0.86
DISCRIMINANT	95.22	93.84	96.05	88.54	90.41	91.45	4.78	0.89

**Table 6:** Brain tumor classification results comparison.

Method	Acc (%)
GLCM + CNN [30]	82.00
Intensity and texture features + PCA + ANN [23]	91.00
BOW + SVM [59]	91.28
2D Discrete Wavelet transform (DWT) and 2D Gabor filter [24]	95.66
SVM+ANN [60]	91.40
Deep CNN+SVM [22]	98.00
Deep Convolution Neural Network VGG 19 + SoftMax classifier [31]	94.58
Capsule networks (CapsNets) [61]	86.56
GLCM + Pre-Trained VGG16 CNN [62]	96.50
GAN + ConvNet (random split) [63]	95.60
**Auto-encoder + KNN (without ACO)**	97.22
**Proposed Method (Auto-encoder + ACO + KNN)**	**99.18**

**Table 7:** COVID-19 classification results comparison. The results that are mentioned in the table are obtained from the literature and the authors used the same dataset that we used for the Brain tumor.

Method	Acc (%)
CNN with data fusion [64]	98.27
ANN [65]	83.98
DNN + SVM [66]	95.33
DNN [67]	94.80
Random Forest [68]	95.90
Stacked-auto-encoder [9]	94.70
Deep Convolutional Auto-encoder [69]	76.52
CNN + Auto-encoder [70]	96.05
Tailored CNN [40]	92.30
Dense Net [41]	88.90
Capsule Networks [71]	95.70
DarkNet-19 based CNN [42]	87.02
Deep Learning [42]	98.08
Deep Learning [43]	86.27
**CNN + SVM (without ACO)**	96.45
**Proposed Method (CNN + ACO + SVM)**	**99.61**

**Table 8:** Comparison between the work of ant colony optimization (ACO) vs. the work of genetic algorithm (GA) as a feature selection (accuracy and time).

Dataset	Combined Methods	ACO			GA		
		Classifiers	Accuracy	Time (h)	Classifiers	Accuracy	Time (h)
**Covid-19**	Auto-encoder	SVM	98.68%	0:27:00	KNN	97.98%	1:00:00
	CNN (AlexNet)	DISCRIMINANT	99.53%	1:17:00	KNN	98.60%	2:07:00
	CNN (GoogleNet)	SVM	98.91%	**0:21:00**	NAÏVE BAYES	98.13%	**0:40:00**
	CNN (ResNet 50)	SVM	**99.61%**	0:43:00	KNN	98.60%	1:05:00
	CNN (DenseNet 201)	NAÏVE BAYES	99.14%	0:39:00	KNN	**98.75%**	1:04:00
**Brain Tumor**	Auto-encoder	KNN	**99.18%**	0:11:00	ENSEMBLE	96.24%	0:16:00
	CNN (AlexNet)	DISCRIMINANT	98.69%	0:12:00	ENSEMBLE	94.61%	0:30:00
	CNN (GoogleNet)	DISCRIMINANT	96.73%	**0:05:00**	KNN	93.96%	**0:09:00**
	CNN (ResNet 50)	KNN	97.87%	0:11:00	ENSEMBLE	**97.06%**	0:16:00
	CNN (DenseNet 201)	SVM	98.20%	0:09:00	ENSEMBLE	96.57%	0:18:00

## Data Availability

It is available in the reference.
